# The impact of a physician-staffed helicopter on outcome in patients admitted to a stroke unit: a prospective observational study

**DOI:** 10.1186/s13049-017-0363-3

**Published:** 2017-02-23

**Authors:** Kamilia S. Funder, Lars S. Rasmussen, Nicolai Lohse, Rasmus Hesselfeldt, Volkert Siersma, Jesper Gyllenborg, Sandra Wulffeld, Ole M. Hendriksen, Freddy K. Lippert, Jacob Steinmetz

**Affiliations:** 1 Department of Anaesthesia, Centre of Head and Orthopaedics 4231 Rigshospitalet, University of Copenhagen, Blegdamsvej 9, DK-2100 Copenhagen, Denmark; 2 The Research Unit for General Practice and Section of General Practice, Department of Public Health, University of Copenhagen, Copenhagen, Denmark; 3 Department of Neurology, Zealand University Hospital Roskilde, Roskilde, Denmark; 4 Prehospital Centre, Region Zealand Slagelse, Denmark; 5 Emergency Medical Services Copenhagen, University of Copenhagen, Copenhagen, Denmark

**Keywords:** Helicopter emergency medical services, Stroke, Mortality, Labour market affiliation, Disability

## Abstract

**Background:**

Transportation by helicopter may reduce time to hospital admission and improve outcome. We aimed to investigate the effect of transport mode on mortality, disability, and labour market affiliation in patients admitted to the stroke unit.

**Methods:**

Prospective, observational study with 5.5 years of follow-up. We included patients admitted to the stroke unit the first three years after implementation of a helicopter emergency medical services (HEMS) from a geographical area covered by both the HEMS and the ground emergency medical services (GEMS). HEMS patients were compared with GEMS patients. Primary outcome was long-term mortality after admission to the stroke unit.

**Results:**

Of the 1679 patients admitted to the stroke unit, 1068 were eligible for inclusion. Mortality rates were 9.04 per 100 person-years at risk (PYR) in GEMS patients and 9.71 per 100 PYR in HEMS patients (IRR = 1.09, 95% CI 0.79–1.49; *p* = 0.60). The 30-day mortality was 7.4% with GEMS and 7.9% with HEMS (OR = 1.02, CI 0.53–1.96; *p* = 0.96). Incidence rate of involuntary early retirement was 6.97 per 100 PYR and 7.58 per 100 PYR in GEMS and HEMS patients, respectively (IRR = 1.19, CI 0.27–5.26; *p* = 0.81). Work ability after 2 years and time on social transfer payments did not differ between groups. We found no significant difference in mean modified Rankin Scale score after 3 months (2.21 GEMS vs. 2.09 HEMS; adjusted mean difference = −0.20, CI −0.74–0.33; *p* = 0.46).

**Discussion:**

The possible benefit of HEMS for neurological outcome is probably difficult to detect by considering mortality, but for the secondary analyses we had less statistical power as illustrated by the wide confidence intervals.

**Conclusion:**

Helicopter transport of stroke patients was not associated with reduced mortality or disability, nor improved labour market affiliation compared to patients transported by a ground unit.

**Trial registration:**

The study was registered at ClinicalTrials.gov (NCT02576379).

**Electronic supplementary material:**

The online version of this article (doi:10.1186/s13049-017-0363-3) contains supplementary material, which is available to authorized users.

## Background

Stroke is one of the leading causes of death and acquired disability among adults [1] and fifteen million people suffer from stroke each year globally [2]. As treatment regimens improve and secondary medical prevention advances, more patients are expected to survive after stroke, some of them unfortunately with stroke-related cognitive and/or physical impairment [3]. Disability acquired during working age may lead to work loss, reduced self-efficacy, and financial struggle [4]. For society, these outcomes may lead to increased socioeconomic burden because of accumulated medical and social costs plus loss of productive years.

Thrombolysis with tissue plasminogen activator is the preferred choice of reperfusion therapy for ischemic stroke if performed within 4.5 h from symptom onset [5, 6]. Hence the introduction of thrombolysis came with an increased urgency related to transfer of patients to specialised stroke facilities. Time to thrombolysis is highly associated with improved outcome [7–10]; the earlier the treatment, the fewer neurons lost [11]. Unfortunately, only a small fraction of stroke patients are eligible for thrombolysis within the 4.5-h therapeutic time window [12], and one explanation for this small number may be system delays, including transportation time.

Helicopter transport of stroke patients may reduce transport times [13] and facilitate timely thrombolytic therapy for patients living far from primary stroke centre facilities [14].

Implementation of the first Danish physician-staffed helicopter emergency medical services (HEMS) in 2010 was associated with increased time to specialised care for stroke patients transported by HEMS compared to patients transported by ground ambulance (ground emergency medical services (GEMS)); however, mortality and degree of disability at 3 months were slightly lower in HEMS patients, though not statistically significantly [15].

The aim of this study was to investigate the effect of transport mode on mortality, disability, and labour market affiliation in patients admitted to the stroke unit at Zealand University Hospital Roskilde during the first 3 years after implementation of HEMS. We hypothesised that stroke patients transported by HEMS would have reduced long-term mortality compared to patients transported by GEMS.

## Methods

### Study design and setting

This single-centre, prospective, observational study had up to 5.5 years of follow-up. Emergency calls in the eastern part of Denmark are answered by a centrally located coordination centre. Depending on triage, expected driving distance, and availability, the dispatcher on call will dispatch one of three means of transportation: 1) a primary ambulance (GEMS) with two emergency medical services (EMS) providers (either emergency medical technician (EMT) providing basic life support or paramedics providing advanced life support); 2) a mobile emergency care unit (MECU) staffed by a physician or a certified nurse anaesthetist and a paramedic; or since May 2010, 3) a HEMS attended by a physician, a pilot, and a paramedic at all times. The physician staffing the MECU and HEMS units was an anaesthesiologist specialised in intensive care and advanced prehospital treatment. All MECU and most HEMS missions are accompanied by a primary ambulance on-scene. The MECU was discontinued in most of the Region Zealand in March 2011.

During the study period, primary ambulance units operated 24 h a day, 7 days a week while the HEMS operated only during daylight hours. Missions included both inter-hospital transfers and transport from out-of-hospital locations.

The stroke centre at Zealand University Hospital Roskilde is the Region Zealand’s sole stroke centre. The centre covers a referral area of approximately 7000 km^2^ with a population of 820,000. Suspected stroke patients were referred to the stroke centre based on a structured telephone visitation by the attending stroke neurologist.

### Selection of participants

We included all patients arriving at the regional stroke unit at Zealand University Hospital Roskilde with suspected acute ischemic stroke, from within the geographical area covered by both HEMS and GEMS. In order to describe the actual stroke population arriving at the regional stroke centre by either HEMS or GEMS, we also included suspected stroke patients from local hospitals within the geographical area.

The geographical catchment area was defined as the area from where HEMS operated during the first year of implementation [15]. However, based on results of an initial study of HEMS [15], the dispatch protocol for the HEMS was changed on January 20, 2012, to allocate HEMS only to the most distant parts of the catchment area.

The 40-month period consisted of the initial 16-month period from the first HEMS study [15] plus an additional 24 months. For patients with multiple contacts, only the first contact was included in the analyses. Patients without emergency medical services (EMS) data (i.e., data on mode of transportation) were excluded. For labour market analyses, we excluded patients not in full-time employment 3 weeks prior to admission.

### Exposure

We compared patients transported by HEMS in a 36-month period (May 1, 2010–April 30, 2013) with patients transported by GEMS in a 40-month period (January 1, 2010–April 30, 2013). In order to obtain data on a cohort of patients before the prehospital system changes we included GEMS patients from a four-month period before the implementation of HEMS.

### Data and data sources

#### Stroke data

The Danish Clinical Registries supervise and authorise usage of national stroke data. The Stroke database (The Danish stroke registry [16]) includes information on demographics, co-morbidity, mRS score at 3 months, National Institute of Health Stroke Scale (NIHSS) score, time of symptom onset, and prehospital, in-hospital, and procedure-specific time intervals.

The mRS score is a widely used and validated measure to assess the degree of global disability and functional independence over time in patients who have suffered a stroke or other neurological illness [17]. The score ranges from 0–6; 0 is scored for no symptoms at all, 5 indicates disability requiring constant care for all needs, and 6 is death. The assessor chooses which questions to ask, and patients are told to refer to pre-stroke functions. The NIHSS score is another commonly used assessment tool to quantify the severity of impairment following a stroke. It consists of 11 items that evaluate different functional abilities, and scores are summed to a total NIHSS score from 0–42, with higher scores indicating increasing severity of stroke. NIHSS score on admission has been shown to be highly correlated with outcome after acute ischemic stroke [18].

At the stroke unit at Zealand University Hospital Roskilde, only patients who receive thrombolysis are accepted for a 3-month follow-up assessment by a neurologist. Hence, assessment of long-term disability defined by the mRS score after 3 months was limited to this group of patients. All health personnel who performed NIHSS and mRS evaluations in this study, were certified in the use of these clinical scales.

### EMS data

Data on geographic location, demographics, mode of transportation, and specific transport time intervals were extracted from either EMS databases at the prehospital centres in Region Zealand (GEMS data) and EMS Copenhagen (HEMS data) or from hospital records in case of missing data.

### Vital status

The Danish Civil Registration System (DCRS) maintains daily updates on vital status and emigration on all persons who have residence in Denmark. Information is linked via the unique Civil Personal Registration number assigned to all residents [19].

### Labour market data (the DREAM database)

Employment status was obtained from the DREAM (Danish Register for Evaluation of Marginalisation) database administered by the Danish Agency of Labour Market and Recruitment. The DREAM database contains data on all social transfer payments such as sickness benefits, unemployment benefits, and pensions (disability and old-age pension) in both the public and private sectors. Citizens who receive social transfer payments are registered on a weekly basis with a code corresponding to the person’s current employment status.

Involuntary early retirement may be granted if work capacity is considered to be permanently reduced to an extent that makes self-reliance impossible. The citizen must be between 18 and 64 years of age, and the application process may take from a few months up to 2 years. All Danish residents are entitled to old-age pension by the time they reach age 65 years (statutory retirement age at the time of the study).

### Outcome measures

The primary outcome was mortality rate after admission to the stroke unit. Secondary outcomes were 30-day mortality, mRS at 3 months, time to involuntary early retirement, prevalence of reduced work ability after 2 years, and percentage of time on social transfer payments during the first 2 years after admission to the stroke centre.

### Statistical analyses

Continuous variables were reported as medians and interquartile ranges (IQR). Categorical data were reported as numbers and percentages (%). Unadjusted comparisons of groups were made using the Mann–Whitney *U* test or Chi-square test where appropriate. We considered *P* values <0.05 as statistically significant. SAS 9.4 statistics (SAS Institute Inc., Cary, NC, USA) was used for statistical analyses.

Time to death was visualised by Kaplan–Meier curves. Mortality rates were computed for GEMS and HEMS and compared in Cox proportional hazard regression models adjusted for relevant covariates. Because of varying access to information on covariates depending on the diagnosis and treatment of the patient, mortality analyses were performed in the whole study population and in two subgroups.

The study population for our primary analysis consisted of all patients admitted to the stroke unit under suspicion of stroke and was not limited to patients who subsequently underwent thrombolysis. This population allowed us to study the effect of prehospital decision making with regard to transport mode. Upon arrival to the hospital, these persons were triaged by the attending stroke neurologist, and some were found not to fulfil criteria for thrombolysis, for example because they suffered from non-ischemic stroke, the therapeutic time window was exceeded or they had multiple comorbidities which did not allow for the patient to undergo thrombolysis.

The first subgroup involved only patients who after admission to the stroke unit were diagnosed with stroke. The second subgroup consisted of patients with stroke who underwent thrombolysis.

The risk of death at 30 days was compared between HEMS and GEMS patients by logistic regression, and adjusted for the same potential confounders as described in the primary outcome analysis.

Labour market analyses included patients diagnosed with stroke, adjusted accordingly for covariates. Incidence rates of involuntary early retirement were computed for HEMS and GEMS and compared by Cox proportional hazard regression models. The prevalence of reduced work ability, as well as the percentage of time on social transfer payments 2 years after admission to the stroke unit, was also compared between HEMS and GEMS patients but assessed by logistic regression models adjusted for sex, age, and co-morbidity. Work ability 2 years after the ischemic stroke was divided into either full work ability or reduced work ability. The percentage of time on social transfer payments was dichotomised into more than 50% and less than/equal to 50%.

Disability (mRS) after 3 months was assessed only in patients who underwent thrombolysis. The mRS was compared between HEMS patients and GEMS patients and analysed by linear regression models, adjusted for sex, age, co-morbidity, and NIHSS at baseline.

### Sensitivity analysis

Additional analyses were performed on the subgroup of patients who were directly referred to the hospital (i.e., not including inter-hospital transferred patients) and who in fact underwent thrombolysis. In the initial study of HEMS implementation [15], time from triaging neurologist contact until arrival at the stroke centre and distance were significantly shorter in the GEMS group; hence, we sought to assess if this factor had changed over the data collection period.

A sensitivity analysis on mortality rate was also performed on patients with transport distances longer than the median distance in the GEMS group.

Finally, we repeated the analyses of all outcomes with adjustment also for transport distance.

### Sample size consideration

We performed a sample size calculation based on the results from the 1-years mortality analysis from initial study of HEMS [15]. We extended the inclusion period by 2 years as well as focused on long-term mortality beyond one year, and based on the calculation we estimated a need for approximately one thousand patients.

We included all eligible patients within our planned inclusion period of 40 months.

## Results

**Fig. 1:**
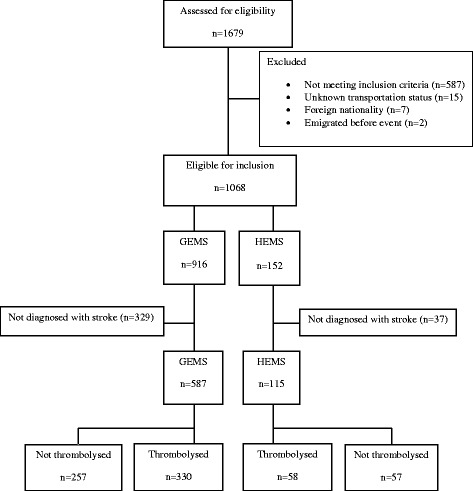
Flowchart of included patients. GEMS: ground emergency medical services; HEMS: helicopter emergency medical services

Of the 1679 patients admitted to the stroke unit during the 40 months of inclusion, 1068 patients (64%) were eligible for inclusion in the study, of whom 702 patients (66%) were diagnosed with stroke (64% (587/916) of GEMS patients and 76% (115/152) of HEMS patients). Thrombolysis was performed in 36% of GEMS (*n* = 330) and 38% of HEMS patients (*n* = 58) (Fig. 1).

Patient characteristics are listed in Table 1.

**Table 1:** Patient characteristics. All patients admitted to the stroke unit

	GEMS (*n* = 916)	HEMS (*n* = 152)	Total (*n* = 1068)	Missing	*P* value
Sex, n (%)				0	0.60
Female	386 (42.1)	68 (44.7)	454 (42.5)		
Male	530 (57.9)	84 (55.3)	614 (57.5)		
Age, median (IQR)	67.9 (56.3–77.7)	69.8 (61.5–76.8)	68.2 (56.7–77.6)	0	0.22
Age, n (%)				0	0.04
< 18 years	6 (0.7)	0 (0.0)	6 (0.6)		
18–60 years	304 (33.2)	36 (23.7)	340 (31.8)		
≥ 61 years	606 (66.2)	116 (76.3)	722 (67.6)		
Inter-hospital transfer, n (%)				0	0.001
No	759 (82.9)	141 (92.8)	900 (84.3)		
Yes	157 (17.1)	11 (7.2)	168 (15.7)		
Diagnosed with stroke, n (%)				0	0.006
No	329 (35.9)	37 (24.3)	366 (34.3)		
Yes	587 (64.1)	115 (75.7)	702 (65.7)		
Thrombolysis, n (%)				0	0.65
No	586 (64.0)	94 (61.8)	680 (63.7)		
Yes	330 (36.0)	58 (38.2)	388 (36.3)		
Full-time work, n (%)				0	0.47
No	578 (63.1)	101 (66.5)	679 (63.6)		
Yes	338 (36.9)	51 (33.5)	389 (36.4)		
Reduced work ability, n (%)				0	0.50
Full work ability	371 (40.5)	56 (36.8)	427 (40.0)		
Reduced work ability	45 (4.9)	7 (4.6)	52 (4.9)		
Involuntary early retirement	72 (7.9)	9 (5.9)	81 (7.6)		
Retirement	378 (41.3)	74 (48.7)	452 (42.3)		
Voluntary early retirement	50 (5.5)	6 (4.0)	56 (5.2)		
Time from contact to triaging neurologist until arrival at the stroke centre (min), median (IQR)	50 (40–65)	60 (51–71)	52 (40–67)	235	<0.0001
Distance (km), median (IQR)	63 (46–73)	97 (71–134)	64 (47–80)	177	<0.0001

*GEMS* ground emergency medical services, *HEMS* helicopter emergency medical services, *IQR* interquartile range

Characteristics for patients diagnosed with stroke and for the subgroup of patients who underwent thrombolysis are shown in Additional files 1 and 2.

Study subjects were followed for a median of 39 (IQR 29–52) months.

**Fig. 2:**
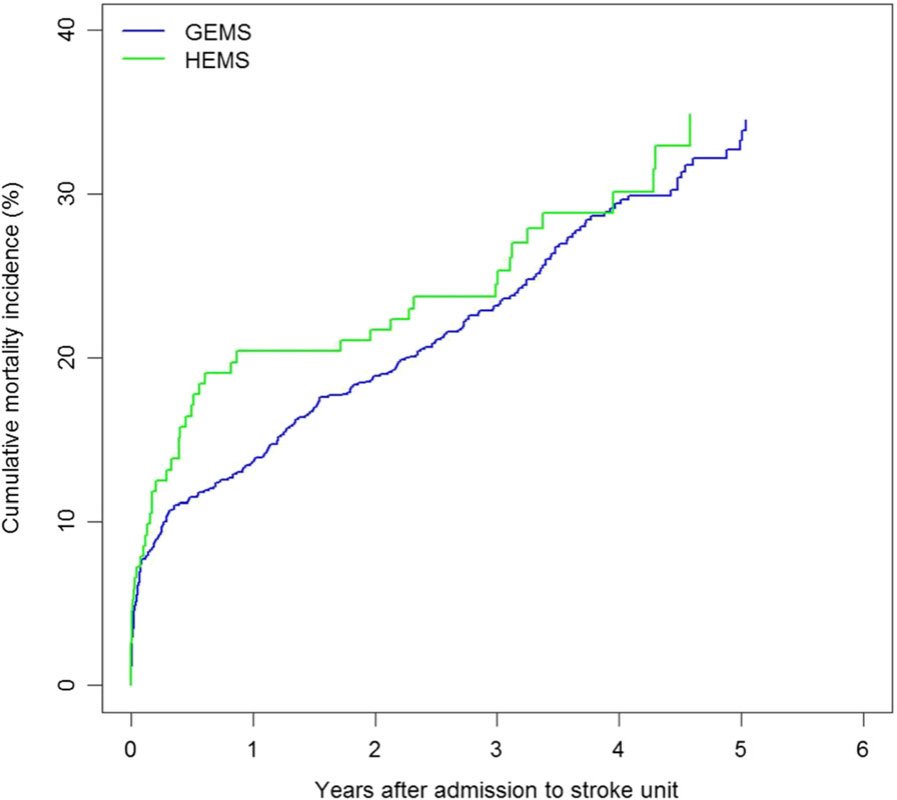
Cumulative risk of death in patients admitted to the stroke unit. GEMS: ground emergency medical services, HEMS: helicopter emergency medical services

Mortality rates for patients admitted to the stroke unit (the whole study population) were 9.04 per 100 person-years at risk (PYR) in GEMS patients and 9.71 per 100 PYR in HEMS patients (Fig. 2) (adjusted incidence rate ratio (IRR) = 1.09, 95% confidence interval (CI) 0.79–1.49; *p* = 0.60) (Table 2).

**Table 2:** Mortality rates and involuntary early retirement in patients admitted to the stroke unit

	Number of persons under observation in each group (GEMS/HEMS)	Number of events	Total PYR	GEMS IR (per 100 PYR)	HEMS IR (per 100 PYR)	Unadjusted IRR (95% CI)	*P* value	Adjusted^a, b, c^IRR (95% CI)	*P* value
**Mortality rates** **(** ***n*** **=** **1068)**	916/152	258/46	2854/474	9.04 (7.94–10.14)	9.71 (6.91–12.52)	1.09 (0.79–1.49)	0.60	1.09 (0.79–1.49)^a^	0.60
Patients diagnosed with stroke (*n* = 702)	587/115	187/40	1773/341	10.55 (9.04–12.06)	11.73 (8.10–15.37)	1.12 (0.79–1.57)	0.53	1.07 (0.75–1.53)^b^	0.72
Patients who underwent thrombolysis (*n* = 388)	330/58	96/16	1031/189	9.31 (7.45–11.17)	8.48 (4.33–12.64)	0.92 (0.54–1.57)	0.92	0.89 (0.50–1.56)^c^	0.68
**Mortality rates with transport distance >63 km** **(** ***n*** **=** **539)**	441/98	114/36	1375/289	8.29 (6.77–9.81)	12.44 (8.37–16.50)	1.49 (1.02–2.16)	0.04	1.36 (0.93–1.98)^a^	0.11
Patients diagnosed with stroke (*n* = 362)	287/75	88/32	864/206	10.18 (8.06–12.31)	15.51 (10.13–20.88)	1.49 (0.99–2.24)	0.05	1.29 (0.84–1.99)^b^	0.25
Patients who underwent thrombolysis (*n* = 202)	167/35	43/12	541/113	7.95 (5.57–10.33)	10.64 (4.62–16.66)	1.33 (0.70–2.52)	0.39	1.03 (0.51–2.07)^c^	0.94
**Mortality rates in directly referred patients**									
Patients who underwent thrombolysis (*n* = 332)	277/55	79/16	871/174	9.07 (7.07–11.07)	9.19 (4.69–13.70)	1.02 (0.60–1.75)	0.94	0.97 (0.55–1.73)^c^	0.92
**Involuntary early retirement**									
Patients diagnosed with stroke (*n* = 101)	89/12	20/3	287/40	6.97 (3.92–10.03)	7.58 (0.00–16.16)	1.12 (0.33–3.77)	0.85	1.19 (0.27–5.26)^b^	0.81
Directly referred patients who underwent thrombolysis (*n* = 44)	37/7	8/0	123/26	6.48 (1.99–10.98)	0.00 (−)	−	−	−	−

*GEMS* ground emergency medical services, *HEMS* helicopter emergency medical services, *PYR* person-years at risk, *IR* incidence rate, *IRR* incidence rate ratio, *CI* confidence interval, *NIHSS* National Institute of Health Stroke Scale. Co-morbidity was defined as having at least one of the following conditions: diabetes, atrial fibrillation, hypertension, previous myocardial infarction, previous stroke

^a^Adjusted for sex (male/female) and age (quadratic)

^b^Adjusted for sex (male/female), age (quadratic), and co-morbidity (yes/no)

^c^Adjusted for sex (male/female), age (quadratic), co-morbidity (yes/no), and NIHSS (continuous)

The 30-day mortality was 7.4% in GEMS patients and 7.9% in HEMS patients (adjusted odds ratio (OR) = 1.02, CI 0.53–1.96; *p* = 0.96) (Table 3).

**Table 3:** 30-day mortality, reduced work ability, and time on social transfer payments for patients admitted to a stroke unit

	Number of persons under observation in each group (GEMS/HEMS)	GEMS, n (%)	HEMS, n (%)	Unadjusted OR (95% CI)	*P* value	Adjusted^a, b, c^OR (95% CI)	*P* value
**30-day mortality (** ***n*** **=** **1068)**	916/152	68 (7.4)	12 (7.9)	1.07 (0.56–2.03)	0.84	1.02 (0.53–1.96)^a^	0.96
Patients diagnosed with stroke (*n* = 702)	587/115	58 (9.9)	12 (10.4)	1.06 (0.55–2.05)	0.86	0.78 (0.37–1.65)^b^	0.51
Patients who underwent thrombolysis (*n* = 388)	330/58	19 (5.8)	3 (5.2)	0.89 (0.26–3.12)	0.86	0.59 (0.12–2.84)^c^	0.51
Directly referred patients who underwent thrombolysis (*n* = 332)	277/55	15 (5.4)	3 (5.5)	1.01 (0.28–3.61)	0.99	0.64 (0.13–3.19)^c^	0.53
**Reduced work ability 2 years after admission to the stroke unit**							
Patients diagnosed with stroke (*n* = 101)	89/12	36 (40.5)	5 (41.7)	1.05 (0.31–3.57)	0.94	0.96 (0.25–3.71)^b^	0.95
Directly referred patients who underwent thrombolysis (*n* = 44)	37/7	17 (46.0)	1 (14.3)	0.20 (0.02–1.79)	0.15	0.16 (0.01–3.38)^c^	0.24
**Over 50% of time on social transfer payments during the 2 years following admission to the stroke unit**							
Patients diagnosed with stroke (*n* = 101)	89/12	40 (44.9)	6 (50.0)	1.23 (0.37–4.09)	0.74	1.13 (0.30–4.31)^b^	0.85
Directly referred patients who underwent thrombolysis (*n* = 44)	37/7	18 (48.7)	2 (28.6)	0.42 (0.07–2.46)	0.34	0.36 (0.03–4.77)^b^	0.44

*GEMS* ground emergency medical services, *HEMS* helicopter emergency medical services, *CI* confidence interval, *OR* odds ratio, *NIHSS* National Institute of Health Stroke Scale. Co-morbidity was defined as having at least one of the following conditions: diabetes, atrial fibrillation, hypertension, previous myocardial infarction, previous stroke

^a^Adjusted for sex (male/female) and age (quadratic)

^b^Adjusted for sex (male/female), age (quadratic), and co-morbidity (yes/no

^c^Adjusted for sex (male/female), age (quadratic), co-morbidity (yes/no), and NIHSS (continuous)

Neither mortality rates nor 30-day mortality differed significantly between GEMS and HEMS patients in any of the other subgroups (Tables 2 and 3).

Incidence rates of involuntary early retirement were 6.97 per 100 PYR and 7.58 per 100 PYR in GEMS and HEMS patients, respectively (adjusted IRR = 1.19, CI 0.27–5.26; *p* = 0.81) (Table 2).

The prevalence of reduced work ability 2 years after the event was 40.5% in GEMS patients and 41.7% in HEMS patients (OR = 0.96, CI 0.25–3.71; *p* = 0.95). The percentage of time (median) on social transfer payments during the first 2 years after admission to the stroke unit was 44.9% (GEMS) vs. 50% (HEMS), with an adjusted OR of receiving social transfer payments for more than half the time of 1.13 (CI 0.30–4.31; *p* = 0.85) (Table 3).

We found no significant difference in mean mRS score after 3 months (2.21 GEMS vs. 2.09 HEMS; adjusted mean difference = −0.20, CI −0.74; 0.33; *p* = 0.46) (Table 4).

**Table 4:** Degree of disability at 3 months defined by the modified Rankin Scale in patients admitted to a stroke unit and who received thrombolysis

	Number of persons under observation in each group (GEMS/HEMS)	GEMS, mean (SD)	HEMS, mean (SD)	Unadjusted mean difference (95% CI)	*P* value	Adjusted^a^ mean difference (95% CI)	*P* value
**Modified Rankin Scale after 3 months**							
Patients who underwent thrombolysis (*n* = 368)	309/57	2.21 (2.07)	2.09 (2.03)	−0.12 (−0.70; 0.46)	0.69	−0.20 (−0.74; 0.33)	0.46
**Modified Rankin Scale after 3 months**							
Directly referred patients who underwent thrombolysis (*n* = 310)	262/54	2.27 (2.10)	2.17 (2.05)	−0.10 (−0.71; 0.51)	0.75	−0.19 (−0.75; 0.38)	0.52

*GEMS* ground emergency medical services, *HEMS* helicopter emergency medical services, *NIHSS* National Institute of Health Stroke Scale. Co-morbidity was defined as having at least one of the following conditions: diabetes, atrial fibrillation, hypertension, previous myocardial infarction, previous stroke

^a^Adjusted for age (quadratic), sex (male/female), co-morbidity (yes/no) and NIHSS (continuous)

None of our sensitivity analyses showed any significant difference between GEMS and HEMS patients (Table 2), and neither did any of the analyses adjusted for transport distance (Additional files 3, 4 and 5). An analysis of possible time gain by HEMS in the group of patients from the most distant areas (>63 km from the hospital), showed inconsistent results. In patients with transport distances between 63–93 km mean transport times were 57 min (GEMS) vs. 56 min (HEMS). With 93–123 km distances times were 75 min vs. 80 min, and distances above 123 km times were 87 min vs. 68 min.

## Discussion

We found that helicopter transport of patients with suspected acute ischemic stroke was not associated with reduced mortality, reduced disability or better labour market affiliation compared to ground transport. The main strengths of the study are the complete inclusion of all suspected stroke patients from a pre-defined geographical area and the complete follow-up in our primary analysis.

Several limitations should be considered when interpreting our results, however. In line with the initial HEMS study [15], we found that HEMS patients in general came from more distant parts of the catchment area, a difference that became even more apparent after the change in dispatch protocol in 2012 when transport distance in the HEMS group increased further. Time from contact with the triaging neurologist to arrival at the hospital was also longer in HEMS patients. In addition to the longer transport distance, this difference may also be related to the fact that HEMS is always dispatched secondarily to a ground unit onsite; thus, response delay is a risk, which may have biased the results in favour of GEMS. Nevertheless, these results did not translate into fewer patients receiving thrombolysis in the HEMS group. Also, because time from contact with the triaging neurologist to arrival at the hospital decreased in both groups (HEMS and GEMS) over the course of the first 3 years despite longer distances in the HEMS group, overall triage and allocation seem to have improved compared with results from the first year of implementation [15].

In addition to the change in protocol, though, our results also suggest another degree of dispatch bias (confounding by indication) because significantly more HEMS patients were diagnosed with stroke, indicating that HEMS may have been allocated to the patients with the most obvious stroke symptoms or that triage by the physician-staffed HEMS was better at identifying patients with stroke compared to the ground unit personnel. Thus, it could be considered a beneficial effect that neither mortality nor labour market affiliation were inferior in HEMS patients.

Hankey et al. [20] found that most early deaths (within 30-day) were directly related to the stroke and subsequent complications like infection and aspiration, whereas long-term mortality mainly was related to other cardiovascular (e.g., cardiac causes, ruptured aortic aneurysms) or non-vascular (e.g., cancer, injuries, suicide) conditions. Therefore, the possible benefits of timely transportation by HEMS for neurological outcome are probably difficult to assess by considering mortality rates.

Labour market affiliation was not significantly different between the groups. Median age in both groups, however, was more than 70 years, and more than two thirds of patients were at or above age 61.

We managed to include the anticipated number of patients for the mortality analyses, but for the secondary analyses we had less statistical power which clearly impedes precision of the results, as illustrated by the wide confidence intervals (Tables 2 and 3). It is therefore not possible to exclude neither a beneficial effect nor a harm of helicopter transport for these endpoints.

Likewise, because mRS after 3 months was evaluated in patients who received thrombolysis, we could assess disability in only one out of three patients (*n* = 368) (Table 4). We found no significant difference between groups in mRS at 3 months, and results were similar in the subgroup analysis of directly referred patients (Table 4). It may be argued that although the mRS is fast and easy to apply, the description of each scale level is subject to some degree of interpretation. Hence, the mRS score is interviewer-dependent, which may reduce reliability and increase the likelihood of overlooking a potential difference. For this reason, mRS may not be sensitive enough to detect small physical changes [21]. However, because patients had a median NIHSS score of 8 before treatment in both groups, there is no reason to expect that disability after 3 months would differ between the groups, either.

The proportion of inter-hospital transfers was much higher during the course of the 3-year data collection period compared to the first-reported study period [15], mainly driven by a high number of GEMS patients (Table 1). The stroke centre at Zealand University Hospital Roskilde is situated close to main highways connecting the hospital to referral hospitals, thus facilitating inter-hospital transportation of GEMS patients. Consequently, it seems that allocation of ground units improved over the course of the 3 years as overall transport time was lower in our study [15].

## Conclusion

We did not find evidence of associations between helicopter transport of stroke patients and reduced mortality, reduced disability, or improved labour market affiliation compared to patients transported by a ground unit.

## Additional files

Additional file 1: Patient characteristics, patients admitted to the stroke unit and diagnosed with stroke. GEMS: ground emergency medical services; HEMS: helicopter emergency medical services; IQR: interquartile range; AMI: acute myocardial infarction. Co-morbidity was defined as having at least one of the following conditions: diabetes, atrial fibrillation, hypertension, previous myocardial infarction, previous stroke. (DOCX 20 kb)
Additional file 2: Patient characteristics, patients admitted to the stroke unit who underwent thrombolysis. GEMS: ground emergency medical services; HEMS: helicopter emergency medical services; IQR: interquartile range; AMI: acute myocardial infarction; NIHSS: National Institute of Health Stroke Scale. Co-morbidity was defined as having at least one of the following conditions: diabetes, atrial fibrillation, hypertension, previous myocardial infarction, previous stroke. (DOCX 20 kb)
Additional file 3: Mortality rates and involuntary early retirement in patients admitted to the stroke unit adjusted for transport distance. GEMS: ground emergency medical services; HEMS: helicopter emergency medical services; PYR: person years at risk; IR: incidence rate; IRR: incidence rate ratio; CI: confidence interval; NIHSS: National Institute of Health Stroke Scale. (DOCX 16 kb)
Additional file 4: 30-day mortality, reduced work ability, and time on social transfer payments for patients admitted to a stroke unit adjusted for transport distance. GEMS: ground emergency medical services; HEMS: helicopter emergency medical services; CI: confidence interval; OR: odds ratio; NIHSS: National Institute of Health Stroke Scale. (DOCX 16 kb)
Additional file 5: Degree of disability at 3 months defined by the modified Rankin Scale in patients admitted to the stroke unit and where thrombolysis was performed adjusted for transport distance. GEMS: ground emergency medical services; HEMS: helicopter emergency medical services; NIHSS: National Institute of Health Stroke Scale. (DOCX 15 kb)

## Abbreviations

CI: Confidence interval
DCRS: The Danish civil registration system
DREAM: Danish register for evaluation of marginalization
EMS: Emergency medical services
EMT: Emergency medical technician
GEMS: Ground emergency medical services
HEMS: Helicopter emergency medical services
IQR: Interquartile range
IR: Incidence rate
IRR: Incidence rate ratio
MECU: Mobile emergency care unit
mRS: Modified rankin scale
NIHSS: National institute of health stroke scale
OR: Odds ratio
PYR: Person-years at risk
SD: Standard deviation
