# Supplementary material for: The impact of a physician-staffed helicopter on outcome in patients admitted to a stroke unit: a prospective observational study
Source: Scand J Trauma Resusc Emerg Med. 2017 Feb 23;25:18. doi: 10.1186/s13049-017-0363-3 (PMC5322627; doi:10.1186/s13049-017-0363-3)
Supplement: Additional file 1: — Patient characteristics, patients admitted to the stroke unit and diagnosed with stroke. GEMS: ground emergency medical services; HEMS: helicopter emergency medical services; IQR: interquartile range; AMI: acute myocardial infarction. Co-morbidity was defined as having at least one of the following conditions: diabetes, atrial fibrillation, hypertension, previous myocardial infarction, previous stroke. (DOCX 20 kb) [file 13049_2017_363_MOESM1_ESM.docx]

|  | **GEMS** | **HEMS** | **Total** | **Missing** | **P value** |
| --- | --- | --- | --- | --- | --- |
|  | (n=587) | (n=115) | (n=702) |  |  |
| **Sex, n (%)** |  |  |  | 0 | 0.46 |
| Female | 223 (38.0) | 48 (41.7) | 271 (38.6) |  |  |
| Male | 364 (62.0) | 67 (58.3) | 431 (61.4) |  |  |
| **Age, median (IQR)** | 70.6 (60.8 ; 79.6) | 72.3 (62.9 ; 78.2) | 70.8 (61.5 ; 79.2) | 0 | 0.70 |
| **Age, n (%)** |  |  |  | 0 | 0.29 |
| <18 years | 2 (0.3) | 0 (0.0) | 2 (0.3) |  |  |
| 18–60 years | 149 (25.4) | 22 (19.1) | 171 (24.4) |  |  |
| ≥61 years | 436 (74.3) | 93 (80.9) | 529 (75.4) |  |  |
| **Inter-hospital transfer, n (%)** |  |  |  | 0 | 0.0040 |
| No | 501 (85.4) | 109 (94.8) | 610 (86.9) |  |  |
| Yes | 86 (14.6) | 6 (5.2) | 92 (13.1) |  |  |
| **Co-morbidity, n (%)** |  |  |  | 33 | 0.74 |
| No | 184 (32.9) | 34 (30.9) | 218 (32.6) |  |  |
| Yes | 375 (67.1) | 76 (69.1) | 451 (67.4) |  |  |
| **Thrombolysis, n (%)** |  |  |  | 0 | 0.26 |
| No | 257 (43.8) | 57 (49.6) | 314 (44.7) |  |  |
| Yes | 330 (56.2) | 58 (50.4) | 388 (55.3) |  |  |
| **Full-time work, n (%)** |  |  |  | 0 | 0.60 |
| No | 376 (64.1) | 77 (67.0) | 453 (64.5) |  |  |
| Yes | 211 (35.9) | 38 (33.0) | 249 (35.5) |  |  |
| **Reduced work ability, n (%)** |  |  |  | 0 | 0.41 |
| Full work ability | 226 (38.5) | 41 (35.7) | 267 (38.0) |  |  |
| Reduced work ability | 15 (2.6) | 5 (4.3) | 20 (2.9) |  |  |
| Involuntary early retirement | 40 (6.8) | 4 (3.5) | 44 (6.3) |  |  |
| Retirement | 267 (45.5) | 59 (51.3) | 326 (46.4) |  |  |
| Voluntary early retirement | 39 (6.6) | 6 (5.2) | 45 (6.4) |  |  |
| **Diabetes, n (%)** |  |  |  | 14 | 0.15 |
| No | 517 (89.9) | 107 (94.7) | 624 (90.7) |  |  |
| Yes | 58 (10.1) | 6 (5.3) | 64 (9.3) |  |  |
| **Atrial fibrillation, n (%)** |  |  |  | 14 | 0.07 |
| No | 474 (82.4) | 101 (89.4) | 575 (83.6) |  |  |
| Yes | 101 (17.6) | 12 (10.6) | 113 (16.4) |  |  |
| **Hypertension, n (%)** |  |  |  | 16 | 0.21 |
| No | 268 (46.6) | 44 (39.6) | 312 (45.5) |  |  |
| Yes | 307 (53.4) | 67 (60.4) | 374 (54.5) |  |  |
| **Previous AMI, n (%)** |  |  |  | 20 | 0.48 |
| No | 514 (90.3) | 105 (92.9) | 619 (90.8) |  |  |
| Yes | 55 (9.7) | 8 (7.1) | 63 (9.2) |  |  |
| **Previous stroke, n (%)** |  |  |  | 14 | 0.90 |
| No | 460 (80.0) | 91 (80.5) | 551 (80.1) |  |  |
| Yes | 115 (20.0) | 22 (19.5) | 137 (19.9) |  |  |
| **Time from contact to triaging neurologist until arrival at the stroke centre (min), median (IQR)** | 50 (40–65) | 62 (54–71) | 52 (40–67) | 118 | <0.0001 |
| **Distance (km), median (IQR)** | 63 (46–73) | 96 (68–134) | 64 (47–80) | 115 | <0.0001 |

Additional file 1. Patient characteristics, patients admitted to the stroke unit and diagnosed with stroke. GEMS: ground emergency medical services; HEMS: helicopter emergency medical services; IQR: interquartile range; AMI: acute myocardial infarction. Co-morbidity was defined as having at least one of the following conditions: diabetes, atrial fibrillation, hypertension, previous myocardial infarction, previous stroke.
