# Supplementary material for: The impact of a physician-staffed helicopter on outcome in patients admitted to a stroke unit: a prospective observational study
Source: Scand J Trauma Resusc Emerg Med. 2017 Feb 23;25:18. doi: 10.1186/s13049-017-0363-3 (PMC5322627; doi:10.1186/s13049-017-0363-3)
Supplement: Additional file 2: — Patient characteristics, patients admitted to the stroke unit who underwent thrombolysis. GEMS: ground emergency medical services; HEMS: helicopter emergency medical services; IQR: interquartile range; AMI: acute myocardial infarction; NIHSS: National Institute of Health Stroke Scale. Co-morbidity was defined as having at least one of the following conditions: diabetes, atrial fibrillation, hypertension, previous myocardial infarction, previous stroke. (DOCX 20 kb) [file 13049_2017_363_MOESM2_ESM.docx]

|  | **GEMS** | **HEMS** | **Total** | **Missing** | **P value** |
| --- | --- | --- | --- | --- | --- |
|  | (n=330) | (n=58) | (n=388) |  |  |
| **Sex, n (%)** |  |  |  | 0 | 0.87 |
| Female | 129 (39.1) | 22 (37.9) | 151 (38.9) |  |  |
| Male | 201 (60.9) | 36 (62.1) | 237 (61.1) |  |  |
| **Age, median (IQR)** | 71.2 (60.9 ; 79.3) | 71.9 (62.9 ; 76.8) | 71.4 (61.7 ; 79.1) | 0 | 0.90 |
| **Age, n (%)** |  |  |  | 0 | 0.37 |
| <18 years | 2 (0.6) | 0 (0.0) | 2 (0.5) |  |  |
| 18–60 years | 82 (24.9) | 10 (17.2) | 92 (23.7) |  |  |
| ≥61 years | 246 (74.5) | 48 (82.8) | 294 (75.8) |  |  |
| **Inter-hospital transfer, n (%)** |  |  |  | 0 | 0.03 |
| No | 277 (83.9) | 55 (94.8) | 332 (85.6) |  |  |
| Yes | 53 (16.1) | 3 (5.2) | 56 (14.4) |  |  |
| **NIHSS before treatment, median (IQR)** | 8.0 (4.0 ; 12.0) | 8.0 (5.0 ; 12.5) | 8.0 (4.0 ; 12.0) | 19 | 0.41 |
| **Modified Rankin Scale score, median (IQR)** | 2.0 (1.0 ; 3.0) | 2.0 (1.0 ; 3.0) | 2.0 (1.0 ; 3.0) | 22 | 0.72 |
| **Full-time work, n (%)** |  |  |  | 0 | 0.97 |
| No | 217 (65.8) | 38 (65.5) | 255 (65.7) |  |  |
| Yes | 113 (34.2) | 20 (34.5) | 133 (34.3) |  |  |
| **Reduced work ability, n (%)** |  |  |  | 0 | 0.32 |
| Full work ability | 122 (37.0) | 21 (36.2) | 143 (36.9) |  |  |
| Reduced work ability | 10 (3.0) | 1 (1.7) | 11 (2.8) |  |  |
| Involuntary early retirement | 21 (6.4) | 0 (0.0) | 21 (5.4) |  |  |
| Retirement | 155 (47.0) | 31 (53.5) | 186 (47.9) |  |  |
| Voluntary early retirement | 22 (6.7) | 5 (8.6) | 27 (7.0) |  |  |
| **Co-morbidity, n (%)** |  |  |  | 14 | 0.55 |
| No | 108 (34.1) | 22 (38.6) | 130 (34.8) |  |  |
| Yes | 209 (65.9) | 35 (61.4) | 244 (65.2) |  |  |
| **Diabetes, n (%)** |  |  |  | 8 | 0.20 |
| No | 293 (91.0) | 56 (96.5) | 349 (91.8) |  |  |
| Yes | 29 (9.0) | 2 (3.5) | 31 (8.2) |  |  |
| **Atrial fibrillation, n (%)** |  |  |  | 7 | 0.59 |
| No | 259 (80.2) | 49 (84.5) | 308 (80.8) |  |  |
| Yes | 64 (19.8) | 9 (15.5) | 73 (19.2) |  |  |
| **Hypertension, n (%)** |  |  |  | 8 | 0.57 |
| No | 156 (48.3) | 30 (52.6) | 186 (49.0) |  |  |
| Yes | 167 (51.7) | 27 (47.4) | 194 (51.0) |  |  |
| **Previous AMI, n (%)** |  |  |  | 11 | 0.20 |
| No | 289 (90.6) | 56 (96.5) | 32 (8.5) |  |  |
| Yes | 30 (9.4) | 2 (3.5) | 345 (91.5) |  |  |
| **Previous stroke, n (%)** |  |  |  | 6 | 0.96 |
| No | 269 (83.0) | 48 (82.8) | 317 (83.0) |  |  |
| Yes | 55 (17.0) | 10 (17.2) | 65 (17.0) |  |  |
| **Time from contact to triaging neurologist until arrival at the stroke centre (min), median (IQR)** | 50 (39–64) | 60 (52–71) | 51 (40–65) | 14 | 0.0002 |
| **Distance (km), median (IQR)** | 63 (46–72) | 99 (66–134) | 64 (48–77) | 61 | <0.0001 |

Additional file 2. Patient characteristics, patients admitted to the stroke unit who underwent thrombolysis. GEMS: ground emergency medical services; HEMS: helicopter emergency medical services; IQR: interquartile range; AMI: acute myocardial infarction; NIHSS: National Institute of Health Stroke Scale. Co-morbidity was defined as having at least one of the following conditions: diabetes, atrial fibrillation, hypertension, previous myocardial infarction, previous stroke.
