# Supplementary material for: The impact of a physician-staffed helicopter on outcome in patients admitted to a stroke unit: a prospective observational study
Source: Scand J Trauma Resusc Emerg Med. 2017 Feb 23;25:18. doi: 10.1186/s13049-017-0363-3 (PMC5322627; doi:10.1186/s13049-017-0363-3)
Supplement: Additional file 3: — Mortality rates and involuntary early retirement in patients admitted to the stroke unit adjusted for transport distance. GEMS: ground emergency medical services; HEMS: helicopter emergency medical services; PYR: person years at risk; IR: incidence rate; IRR: incidence rate ratio; CI: confidence interval; NIHSS: National Institute of Health Stroke Scale. (DOCX 16 kb) [file 13049_2017_363_MOESM3_ESM.docx]

|  | **Number of persons under observation in each group (GEMS/HEMS)** | **Number of events** | **Total PYR** | **GEMS IR**  **(per 100 PYR)** | **HEMS IR**  **(per 100 PYR)** | **Unadjusted IRR (95%CI)** | **P value** | **Adjusted^1,2,3^ IRR (95%CI)** | **P value** | **Adjusted IRR (95%CI) also for distance (squared)** | **P value** |
| --- | --- | --- | --- | --- | --- | --- | --- | --- | --- | --- | --- |
| **Long-term mortality (n=1068)** | 916/152 | 258/46 | 2854/474 | 9.04 (7.94-10.14) | 9.71 (6.91-12.52) | 1.09 (0.79-1.49) | 0.60 | 1.09 (0.79-1.49)^1^ | 0.60 | 1.27 (0.87-1.84) | 0.21 |
| patients diagnosed with stroke (n=702) | 587/115 | 187/40 | 1773/341 | 10.55 (9.04-12.06) | 11.73 (8.10-15.37) | 1.12 (0.79-1.57) | 0.53 | 1.07 (0.75-1.53)^2^ | 0.72 | 1.16 (0.76-1.78) | 0.49 |
| patients who underwent thrombolysis (n=388) | 330/58 | 96/16 | 1031/189 | 9.31 (7.45-11.17) | 8.48 (4.33-12.64) | 0.92 (0.54-1.57) | 0.92 | 0.89 (0.50-1.56)^3^ | 0.68 | 0.95 (0.48-1.87) | 0.88 |
| **Involuntary early retirement** |  |  |  |  |  |  |  |  |  |  |  |
| patients diagnosed with stroke (n=101) | 89/12 | 20/3 | 287/40 | 6.97 (3.92-10.03) | 7.58 (0.00-16.16) | 1.12 (0.33-3.77) | 0.85 | 0.84 (0.19-3.73)^2^ | 0.81 | 0.62 (0.11-3.48) | 0.59 |
| ^1^Adjusted for sex and age  ^2^Adjusted for sex, age, and co-morbidity  ^3^Adjusted for sex, age, co-morbidity, and NIHSS | | | | | | | | | | | |

Additional file 3. Mortality rates and involuntary early retirement in patients admitted to the stroke unit adjusted for transport distance. GEMS: ground emergency medical services; HEMS: helicopter emergency medical services; PYR: person years at risk; IR: incidence rate; IRR: incidence rate ratio; CI: confidence interval; NIHSS: National Institute of Health Stroke Scale.
