# Supplementary material for: The impact of a physician-staffed helicopter on outcome in patients admitted to a stroke unit: a prospective observational study
Source: Scand J Trauma Resusc Emerg Med. 2017 Feb 23;25:18. doi: 10.1186/s13049-017-0363-3 (PMC5322627; doi:10.1186/s13049-017-0363-3)
Supplement: Additional file 4: — 30-day mortality, reduced work ability, and time on social transfer payments for patients admitted to a stroke unit adjusted for transport distance. GEMS: ground emergency medical services; HEMS: helicopter emergency medical services; CI: confidence interval; OR: odds ratio; NIHSS: National Institute of Health Stroke Scale. (DOCX 16 kb) [file 13049_2017_363_MOESM4_ESM.docx]

|  | **Number of persons under observation in each group (GEMS/HEMS)** | **GEMS, n (%)** | **HEMS, n (%)** | **Unadjusted OR (95%CI)** | **P value** | | **Adjusted^1,2,3^ OR (95%CI)** | **P value** | **Adjusted OR (95%CI) also for distance (squared)** | **P value** |
| --- | --- | --- | --- | --- | --- | --- | --- | --- | --- | --- |
| **30-day mortality (n=1068)** | 916/152 | 68 (7.4) | 12 (7.9) | 1.07 (0.56-2.03) | 0,84 | 1.02 (0.53-1.96)^1^ | | 0.96 | 1.30 (0.61-2.79) | 0.49 |
| patients diagnosed with stroke (n=702) | 587/115 | 58 (9.9) | 12 (10.4) | 1.06 (0.55-2.05) | 0,86 | 0.78 (0.37-1.65)^2^ | | 0.51 | 1.12 (0.47-2.70) | 0.80 |
| patients who underwent thrombolysis (n=388) | 330/58 | 19 (5.8) | 3 (5.2) | 0.89 (0.26-3.12) | 0,86 | 0.59 (0.12-2.84)^3^ | | 0.51 | 0.91 (0.16-5.18) | 0.92 |
| **Reduced work ability two years after admission to the stroke unit** |  |  |  |  |  |  | |  |  |  |
| patients diagnosed with stroke (n=101) | 89/12 | 36 (40.5) | 5 (41.7) | 1.05 (0.31-3.57) | 0,94 | 0.96 (0.25-3.71)^2^ | | 0.95 | 0.53 (0.09-3.30) | 0.50 |
| **Over 50% of time on social transfer payments during the two years following admission to the stroke unit** |  |  | |  |  |  | |  |  |  |
| patients diagnosed with stroke (n=101) | 89/12 | 40 (44.9) | 6 (50.0) | 1.23 (0.37-4.09) | 0,74 | 1.13 (0.30-4.31)^2^ | | 0.85 | 0.67 (0.11-4.21) | 0.67 |
| ^1^Adjusted for sex and age  ^2^Adjusted for sex, age, and co-morbidity  ^3^Adjusted for sex, age, co-morbidity, and NIHSS | | | | | | | | | | |

Additional file 4. 30-day mortality, reduced work ability, and time on social transfer payments for patients admitted to a stroke unit adjusted for transport distance. GEMS: ground emergency medical services; HEMS: helicopter emergency medical services; CI: confidence interval; OR: odds ratio; NIHSS: National Institute of Health Stroke Scale.
