# Supplementary material for: The impact of a physician-staffed helicopter on outcome in patients admitted to a stroke unit: a prospective observational study
Source: Scand J Trauma Resusc Emerg Med. 2017 Feb 23;25:18. doi: 10.1186/s13049-017-0363-3 (PMC5322627; doi:10.1186/s13049-017-0363-3)
Supplement: Additional file 5: — Degree of disability at 3 months defined by the modified Rankin Scale in patients admitted to the stroke unit and where thrombolysis was performed adjusted for transport distance. GEMS: ground emergency medical services; HEMS: helicopter emergency medical services; NIHSS: National Institute of Health Stroke Scale. (DOCX 15 kb) [file 13049_2017_363_MOESM5_ESM.docx]

|  | **Number of persons under observation in each group (GEMS/HEMS)** | **GEMS, mean (SD)** | **HEMS, mean (SD)** | **Unadjusted mean difference (95%CI)** | **P value** | **Adjusted^1^ mean difference (95%CI)** | **P value** | **Adjusted mean difference (95%CI) also for distance (squared)** | **P value** |
| --- | --- | --- | --- | --- | --- | --- | --- | --- | --- |
| **Modified Rankin Scale (mRS)** |  |  |  |  |  |  |  |  |  |
| patients who underwent thrombolysis (n=368) | 309/57 | 2.21 (2.07) | 2.09 (2.03) | -0.12 (-0.70 ; 0.46) | 0.69 | -0.20 (-0.74 ; 0.33) | 0.46 | -0.02 (-0.69 ; 0.66) | 0.96 |
| ^1^Adjusted for age, sex, co-morbidity and NIHSS. | | | | | | | | | |

Additional file 5. Degree of disability at 3 months defined by the modified Rankin Scale in patients admitted to the stroke unit and where thrombolysis was performed adjusted for transport distance. GEMS: ground emergency medical services; HEMS: helicopter emergency medical services; NIHSS: National Institute of Health Stroke Scale.
